# Cognitive impairments in autoimmune encephalitis: the role of autoimmune antibodies and oligoclonal bands

**DOI:** 10.3389/fimmu.2024.1405337

**Published:** 2024-09-27

**Authors:** Ayal Rozenberg, Shahar Shelly, Adi Vaknin-Dembinsky, Tal Friedman-Korn, Tal Benoliel-Berman, Polina Spector, Natalya Yarovinsky, Diana Guber, Lilach Gutter Kapon, Yair Wexler, Esther Ganelin-Cohen

**Affiliations:** ^1^ Department of Neurology, Rambam Health Care Campus, Haifa, Israel; ^2^ Neuroimmunology Laboratory, Ruth and Bruce Rapaport Faculty of Medicine, Technion–Israel Institute of Technology, Haifa, Israel; ^3^ Department of Neurology and Laboratory of Neuroimmunology and Agnes-Ginges Center for Neurogenetics, Faculty of Medicine, Hebrew University of Jerusalem, Jerusalem, Israel; ^4^ Department of Neurology, Carmel Medical Center, Haifa, Israel; ^5^ Multiple Sclerosis Center, Sheba Medical Center, Tel Hashomer, Israel; ^6^ Faculty of Medical and Health Sciences, Tel Aviv University, Tel Aviv, Israel; ^7^ Clinical Immunology and Tissue Typing Laboratory, Rambam Health Care Campus, Haifa, Israel; ^8^ School of Neurobiology, Biochemistry and Biophysics, George S. Wise Faculty of Life Sciences, Tel Aviv University, Tel Aviv, Israel; ^9^ Neuroimmunological Clinic, Institute of Pediatric Neurology, Schneider Children’s Medical Center of Israel, Petah Tikva, Israel

**Keywords:** autoimmune encephalitis (AIE), autoimmune encephalitis antibodies, oligoclonal bands (OCBs), cognitive change, behavioral change, NMDA

## Abstract

**Introduction:**

The presence of oligoclonal bands (OCBs) in cerebrospinal fluid (CSF) is a pivotal diagnostic marker for multiple sclerosis (MS). These bands play a crucial role in the diagnosis and understanding of a wide array of immune diseases. In this study, we explore the relationship between the cognitive profile of autoimmune encephalitis (AIE) and the presence of OCBs in CSF, with a particular emphasis on NMDA receptor antibodies.

**Methods:**

We studied a cohort of 21 patients across five tertiary centers, segregated into two distinct categories. One group comprised individuals who tested positive only for autoimmune encephalitis antibodies indicative of encephalitis, while the other group included patients whose CSF was positive for both autoimmune encephalitis antibodies and OCBs. Our investigation focused primarily on cognitive functions and behavioral alterations, supplemented by auxiliary diagnostic assessments such as CSF cell count, magnetic resonance imaging (MRI), and electroencephalogram (EEG) results, evaluated for the two patient groups. To validate our findings, we employed statistical analyses such as Fisher’s exact test with Benjamini-Hochberg correction.

**Results:**

Our study included 21 patients, comprising 14 who were presented with only autoimmune encephalitis antibodies, and 7 who were dual-positive. Among these patients, we focused on those with NMDA receptor antibodies. Of these, five were dual positive, and nine were positive only for NMDA receptor antibodies. The dual-positive NMDA group, with an average age of 27 ± 16.47 years, exhibited significantly higher CSF cell counts (p=0.0487) and more pronounced language and attention deficits (p= 0.0264). MRI and EEG results did not differ significantly between the groups.

**Conclusions:**

Our results point to OCBs as an additional marker of disease severity in AIE, especially in NMDA receptor-antibody positive patients, possibly indicating a broader inflammatory process, as reflected in elevated CSF lymphocytes. Regular testing for OCBs in cases of suspected AIE may aid in disease prognosis and identification of patients more prone to language and attention disorders, improving diagnosis and targeting treatment for these cognitive aspects.

## 1 Introduction

Autoimmune encephalitis (AIE) presents as a distinct condition, hallmarked by an inflammation of the brain, predominantly within the limbic system. This inflammation is mediated by a spectrum of autoantibodies (1–4). In certain patients, the specific antibodies responsible for the disease remain elusive despite extensive investigation. These individuals may still be diagnosed with AIE if they fulfill the clinical criteria established by Dalmau and Graus (5). Such cases are classified as seronegative AIE, and require evidence of pathological CSF findings, such as the presence of cells or elevated protein levels, and characteristic MRI and EEG findings.

AIE ‘s non-specific manifestations, spanning from agitation to psychosis, pose particular challenges especially in seronegative cases, making definitive diagnoses difficult (4, 6, 7). A precise diagnosis is essential as it dictates the need for early intervention with immunosuppressive therapy. However, the determination of optimal treatment intensity and duration remains a significant challenge due to the current lack of reliable diagnostic biomarkers and prognostic tools. These symptoms, often initial indicators of the disease, underscore the complexity of AIE management, emphasizing the critical need for early and precise diagnosis to guide immunosuppressive treatment strategies.

Oligoclonal bands (OCBs), specific immunoglobulins found in the cerebrospinal fluid (CSF) but not in matching serum samples, suggest intrathecal synthesis is key to diagnosing a range of inflammatory diseases, including autoimmune disorders, central nervous system infections, and neurodegenerative diseases (8, 9). The inclusion of OCBs in the 2017 revised McDonald criteria for diagnosing Multiple Sclerosis (MS) as a laboratory marker was a pivotal development, aiding in the identification of clinical isolated syndromes and meeting criteria for dissemination in time, thus facilitating early diagnosis and better treatment decisions for autoimmune and inflammatory diseases (10, 11).

Blinder and Lewerenz (12) comprehensive examination of cerebrospinal fluid (CSF) in patients with AIE identified distinct subtypes characterized by various autoantibodies, each exhibiting a range of abnormalities. Specifically, diseases linked to GAD antibodies frequently demonstrated a high prevalence of OCBs, while CSF pleocytosis or elevated protein levels were less frequently observed. This distinct pattern suggests a unique subtype of AIE. Additionally, there is evidence indicating the involvement of cytotoxic T cells in the pathogenesis of these conditions, particularly, in those involving intracellular antigens such as GAD. This highlights the diverse immunological mechanism at play for different AIE subtypes.

Some AIE patients have positive OCBs, although their value in disease diagnosis and prognosis remains unclear. Hébert et al. (13) showed that adding OCBs as a pathologic CSF finding in AIE criteria can significantly reduce the incidence of normal CSF findings, underlining OCBs’ potential diagnostic value. Beyond aiding in AIE diagnosis, OCBs serve as a predictive marker for AIE prognosis. For example, in NMDA encephalitis, the presence of OCBs at the onset was associated with a more refractory disease, prolonged hospitalization, and poorer outcomes, amongst other factors (14).

Cognitive impairment is a hallmark of AIE, manifesting as memory deficits, language disturbances, and attentional dysfunction. This is especially evident in anti-NMDA receptor encephalitis, where antibodies targeting the GluN1 subunit led to receptor under-expression, resulting in cognitive decline, particularly in memory and speech disorders (15). The correlation between cognitive impairments and OCBs highlights the importance of early detection and targeted intervention in AIE. Studies, including those by Cucuzza et al. (16), document cases where specific autoantibodies, such as anti-AMPA GluR3, cause significant cognitive deficits, reinforcing the need for prompt diagnosis and treatment.

Given their potential prognostic value, we examined whether OCBs could predict more inflammatory involvement or sequelae in patients with AIE, particularly those who are NMDA receptor antibody positive. To this end, we retrospectively studied the clinical manifestations including language. Attention, and behavioral changes, as well as variables such as: imaging, CSF, and EEG finding in AIE patients, with and without OCBs in the CSF at diagnosis.

## 2 Methods

### 2.1 Patient demographics and informed consent

We conducted a retrospective, uncontrolled study by reviewing medical records from five tertiary medical centers (blinded) with ethics approval from the respective institutional review boards. The datasets included MRI and EEG reports, clinical updates, and specific data from Neurology Mosaics (Autoimmune Encephalitis Panel) from selected centers.

Neuro-immunology experts at each center identified 26 patients with AIE based on criteria by Dalmau and Graus (5) and Graus et al. (4), focusing on positive tests for autoimmune antibodies indicative of encephalitis and OCBs. Only patients with a positive autoantibody result in the CSF were included. Out of these, 21 patients were divided into two groups:14 with positive autoimmune encephalitis antibodies and negative OCBs (single positive group **-** [SPG]) and 7 patients positive for both markers (double positive group [DPG]). For the analysis we specifically focused on NMDA receptor antibody-positive patients, with five patients in the DPG and nine patients in the SPG.

### 2.2 Demographic data and tests

Our analysis included demographic data (age, sex), cognitive and behavioral changes due to AIE, CSF cell counts, MRI findings, and EEG results. Change in language and attention were assessed based on the neurologist’s familiarity with the patients and the results from the Montreal Cognitive Assessment (MOCA) or the Mini-Mental State Examination (MMSE) subsections that evaluate language and attention. Behavior changes were evaluated through the neurologist’s interactions with the patient and their close relatives, as well as the need for treatment due to observed behavioral changes. For MRI changes, we considered hyperintense signals on T2-weighted fluid-attenuated inversion recovery (FLAIR) sequences, which were either highly restricted to one or both medial temporal lobes (indicative of limbic encephalitis) or found in multifocal areas involving grey matter, white matter, or both consistent with demyelination or inflammation. EEG criteria were based on the presence of epileptic activity, slow-wave activity involving the temporal lobes, or the appearance of a delta brush pattern appearance.

### 2.3 Data collection and interpretation

The neuroimmunologists at each center collected and interpreted the data based on medical records from their respective institutions, often drawing on their familiarity with the patients. The laboratory techniques were conducted by four out of the five centers and remained consistent. For The Neurology Mosaics panel incorporated glutamate receptors (type NMDA), (type AMPA1 and type AMPA2), as well as contactin-associated protein 2 (CASPR2), leucine-rich glioma-inactivated protein 1 (LGI1), and GABAB receptor (GABARB1/B2), we utilized the Cell-based Immunofluorescence Assay (17). Immunofluorescence to detect anti-mGluR5 antibodies.

In case of positive results, the tests were repeated to ensure accuracy. Sequential dilutions were performed up to 1:100 to enhance reliability and avoid false positive results. OCBs assessment was performed using Hydragel CSF Isofocusing (Sebia Co., France), with two or more exclusive CSF bands considered a positive result.

### 2.4 Statistical analysis

We employed Fisher’s exact test for between-group comparisons, and for CSF cells we used log(x+1) transformation with age and sex as random variables. The False Discovery Rate was applied (18, 19) to adjust for multiple comparisons and control for the false positive rate. The significance level was set at 0.05.

## 3 Results

### 3.1 Demographics and baseline characteristics

**Figure 1:**
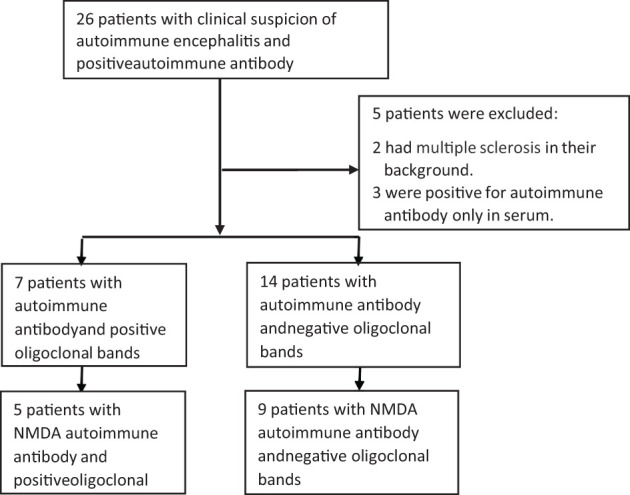
Distribution of Patients with positive AIE antibodies, with and without OCBs. Twenty-six patients were included in the initial screening, with 21 included in the final analysis., Fourteen patients belonged to the SPG, and seven were in the DPG. Of these, we selected five patients with DPG who had both NMDA and OCB and nine patients from the SPG who were solely NMDA positive with negative OCB. CSF, cerebrospinal fluid; DPG, double positive group; SPG, single positive group.

We included in the first screening 26 patients; 21 were eligible after exclusions. Two patients were excluded due to MS in their background and three due to antibodies present only in serum, as shown in **Figure 1**. Detailed information about age, sex, type of associate antibody found positive in CSF, co-morbid tumor and clinical characteristics are shown in **Tables 1**, **2**.

**Table 1:** Characteristics of patients positive for NMDA autoimmune encephalitis antibodies and negative for oligoclonal bands.

Patient Number	Age (years)	Sex	Type of antibody	Associated tumor	Clinical presentation
1	33	Female	NMDA	Dermoid cyst	Memory deficit, other cognitive changes
2	33	Female	NMDA	Ovarian teratoma	Behavioral changes
3	33	Male	NMDA	None	Behavioral changes, memory deficit
4	66	Female	NMDA	None	Behavioral changes, memory deficit
5	22	Female	NMDA	None	Memory deficit, other cognitive changes
6	13	Male	NMDA	None	Behavioral changes
7	65	Male	NMDA	None	Behavioral changes
8	42	Male	NMDA	None	Behavioral changes, memory deficit
9	91	Male	NMDA	None	Behavioral changes, memory deficit

**Table 2:** Characteristics of patients dual-positive for NMDA autoimmune encephalitis antibodies and oligoclonal bands.

	Age (years)	Sex	Type of antibody	Associated tumor	Clinical presentation
1	27	Female	NMDA	None	Memory deficit, attention problems, language problems
2	56	Female	NMDA	Rectal cancer	Behavioral changes, memory deficit, attention problems, language problems
3	22	Female	NMDA	None	Behavioral changes, memory deficit, attention problems
4	16	Female	NMDA	None	Behavioral changes
5	17	Female	NMDA	None	Behavioral changes, memory deficit, language problems

The single positive group (SPG) comprised 14 patients, including 7 females, with an average age of 48 years. The double positive group (DPG) consisted of seven patients, six of whom were female, with an average age of 30 years, (**Table 3**). Tumor associations within the SPG included one case of ovarian teratoma, and one case of dermoid cyst (**Table 1**). The DPG had one case with a rectal cancer tumor (**Table 2**). In **Supplementary Table S1**, we present data on AIE encephalitis patients in the SPG and DPG groups who had antibodies other than NMDA. Due to different mechanisms of action of these antibodies, our main analysis in the text focuses exclusively on NMDA antibodies.

**Table 3:** Demographic, cognitive, behavioral, and CSF characteristics of AIE patients.

Variable		Oligoclonal bands		Overall
		DPG	SPG	
n		7 (33.3%)	14 (66.67%)	21
Sex	Male	1 (12.5%)	7 (87.5%)	8
	Female	6 (46.15%)	7 (53.85%)	13
Age (years), mean ± SD, median, (range)		30 ± 18 22 (17, 56)	48 ± 24 45 (33, 66)	45 ± 24 45 (22, 64)
CSF cells mean ± SD, median, (range)		36.1 ± 42.1 21.0 (12.5, 39.0)	8.5 ± 15.6 2.0 (1.0, 6.0)	16.8 ± 27.2 4.5 (1.0, 19.5)
Language	Impaired	5 (71.43%)	2 (28.57%)	7
	Normal	2 (14.29%)	12 (85.71%)	14
Attention deficit	Yes	3 (100%)	0 (0%)	3
	No	4 (22.22%)	14 (77.78%)	18
Behavioral change	Yes	5 (29.41%)	12 (70.59%)	17
	No	2 (50%)	2 (50%)	4
Memory	Impaired	4 (30.77%)	9 (69.23%)	13
	Normal	3 (37.5%)	5 (62.5%)	8

### 3.2 Auxiliary test results

**Figure 2:**
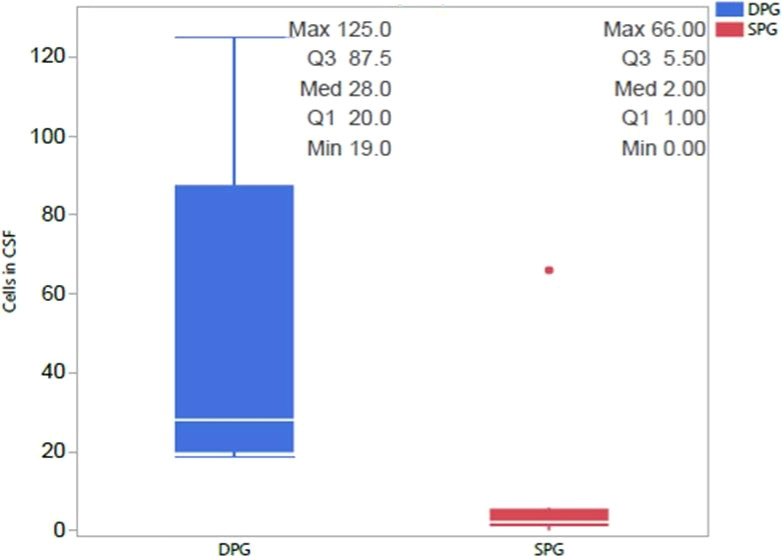
Differences in CSF cell Counts between NMDA-positive SPG and DPG groups. We compared the two groups and observed that the DPG group had significantly more cells in CSF than the SPG group, with this difference remaining significant after correction for multiple comparisons 462 (p=0.0487). CSF, cerebrospinal fluid; DPG, double positive group; SPG, single positive group.

A comparative analysis of CSF, MRI, and EEG results was conducted to identify potential differences between the SPG and the DPG groups of NMDA encephalitis patients. In the CSF analysis the DPG group of NMDA exhibited significantly higher cell counts, averaging 47.40 compared to 9.56 in the SPG group of NMDA, as depicted in **Figure 2** (p= 0.0487). MRI scans did not reveal any significant differences between the groups concerning lesions in the limbic area. EEG results also showed no disparities between the SPG and DPG groups of NMDA.

### 3.3 Cognitive and behavioral results

**Figure 3:**
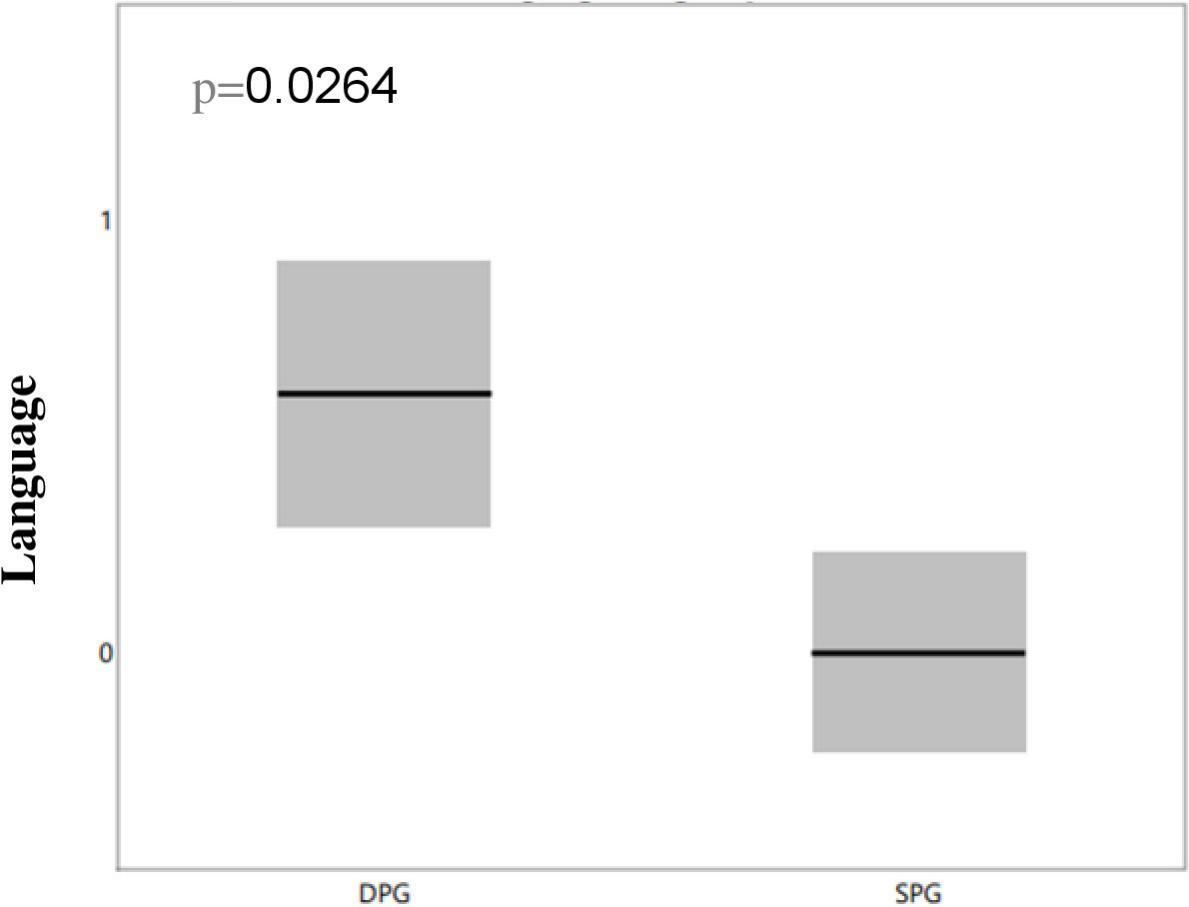
Differences in language deficits between NMDA-positive SPG and DPG groups. We compared language deficits between the two groups and observed that the DPG group had more significant language deficit than the SPG group, with the difference remaining significant after correction for multiple comparisons (p=0.0264). DPG, double positive group; OCBs, oligoclonal bands; SPG, single positive group.

**Figure 4:**
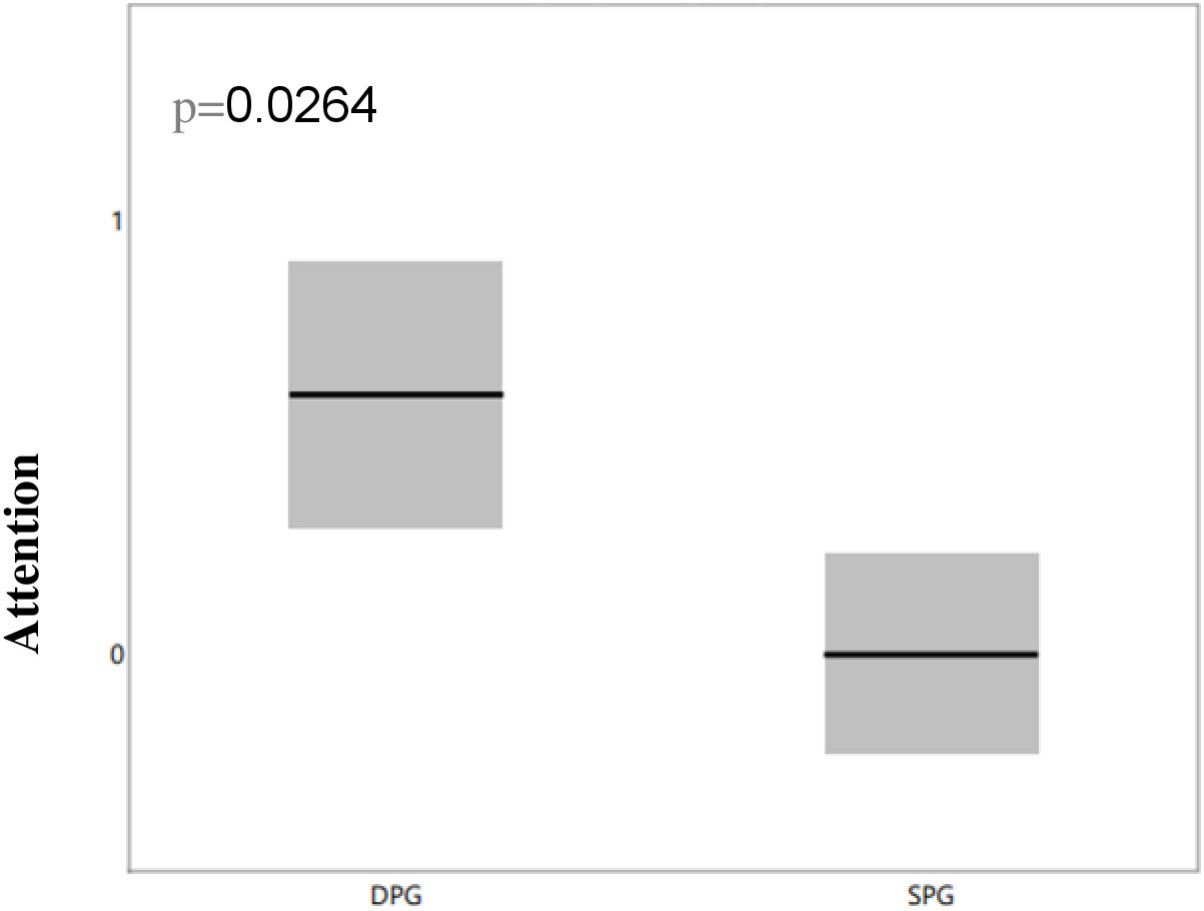
Differences in attention deficit between NMDA-positive SPG and DPG groups. We compared attention deficits between the two groups and observed that the DPG group had more pronounced attention deficits than the SPG group, with the difference remaining significant after correction for multiple comparisons (p=0.0264) DPG, double positive group; OCBs, oligoclonal bands; SPG, single positive group.

The DPG group with NMDA antibodies demonstrated a higher incidence of language deficits (p=0.0264), as highlighted in **Figure 3**, and a greater prevalence of attention impairments (p=0.0264), as shown in **Figure 4**. However, there were no significant differences between the groups regarding memory issues or other behavioral changes.

## 4 Discussion

In this study we explored the role of OCBs in AIE, particularly of the NMDA type, addressing both diagnostic and prognostic challenges due to the complexity of AIE ‘s presentation, which includes diverse cognitive and behavioral changes. Traditionally associated with MS, OCBs have emerged as potential indicators of AIE severity, warranting our focused investigation. Our findings revealed that patients with dual positivity for OCBs and autoimmune encephalitis antibodies (DPG), especially NMDA, exhibited more significant language and attention deficits compared to those positive only for NMDA antibodies (SPG). This suggests a link between the presence of OCBs and specific cognitive impairments in AIE, underscoring the importance of further research into the disease spectrum and OCBs’ diagnostic utility.

Interestingly, the NMDA-positive DPG group also showed higher CSF cell counts, indicating that the presence of OCB is associated with higher levels of inflammation, as OCB itself is an indicator of inflammatory processes in AIE. While MRI and EEG outcomes did not differ significantly between groups, these modalities may be normal in AIE patients, in different clinical presentations (4, 5). This research underscores the importance that an inflammation specific biomarker, such as OCBs, may have in disease diagnosis and prognosis. A study by Ganelin-Cohen et al. (20) implicated OCBs with positive anti-MOG. That study demonstrated a link between OCBs and disease severity in patients with anti-MOG antibodies, highlighting their role in predicting illness severity. Similarly, MS research by Ben Noon et al. (21) revealed that OCBs were associated with higher disease severity, emphasizing the broader relevance of OCBs in autoimmune and inflammatory neurological disorders. A recent study by Xue et al. (22), comparing clinical differences in AIE patients based on OCBs status, reported more severe inflammation in OCBs-positive patients but did not find significant differences in psychiatric disorders, language disorders, or cognitive dysfunction. This finding contrasts with our findings, possibly due to our study’s more focused approach and the meticulous cognitive assessment employed, offering a potential explanation for the observed discrepancies.

Cognitive impairment, a central feature of AIE, was particularly evident in our study. Patients with dual positivity for OCBs and NMDA antibodies exhibited more pronounced deficits in cognitive functions such as language and attention. These findings align with previous research that highlighting the role of autoantibodies in cognitive and neuropsychiatric symptoms (16). In anti-NMDA receptor encephalitis, the presence of antibodies against the GluN1 subunit is known to result in significant cognitive impairments, as the autoimmune attack on synaptic function correlates with observed cognitive deficits.

(15) AIE often leads to cognitive impairments due to immune attacks on glutamate receptors, essential for normal brain function, including learning and memory. The disruption of glutamatergic neurotransmission, caused by antibodies against ionotropic and metabotropic glutamate receptors, interferes with neuronal communication. This disturbance primarily affects the brain regions involved in cognition, resulting in memory deficits and other cognitive issues commonly seen in AIE patients.

Evidence of broader brain damage, such as superficial white matter damage, can harm short-range association fibers and intracortical myelin, manifesting as impairments in attention and memory. Extensive changes in deep white matter integrity also correlates with disease severity (23). The presence of more widespread inflammation, as indicated by OCBs, may account for these changes.

Our study included only patients with positive autoimmune encephalitis antibodies. Seronegative cases were not included to keep out study as “clean” as possible. In addition, we avoided adding another control group and instead focused on the seropositive group and then compared positive and negative OCBs patients. Despite the insights provided, our study faces limitations, notably the small sample size. This limitation, while understandable given AIE ‘s rarity, necessitates cautious interpretation of our findings and highlights the need for further research with larger cohorts. We use a cell-based immunofluorescent assay to identify autoimmune antibodies, accounting for potential false positive results. For positive findings, we repeat the examination and consider only those results confirmed by CSF analysis. Additionally, our diagnostic decisions are not based solely on positive antibody results; we also consider clinical features, evidence of inflammation in CSF, and supportive EEG or MRI findings consistent with AIE. Given this comprehensive approach the likelihood of false positive results is very low.

In summary, our study deepens the understanding of the role of OCBs in AIE, with a specific focus on NMDA receptor associated AIE. The findings suggest that incorporating OCBs analysis into routine clinical evaluations may positively impact diagnostic and interventional strategies in managing these patients. We advocate for the inclusion of OCBs analysis in the assessment of AIE, as it could enhance diagnostic precision and patient outcomes.

## Data Availability

The original contributions presented in the study are included in the article/**Supplementary Material**. Further inquiries can be directed to the corresponding author.
