# Supplementary material for: Cognitive impairments in autoimmune encephalitis: the role of autoimmune antibodies and oligoclonal bands
Source: Front Immunol. 2024 Sep 27;15:1405337. doi: 10.3389/fimmu.2024.1405337 (PMC11472350; doi:10.3389/fimmu.2024.1405337)
Supplement: Supplementary file 1 [file Table1.docx]

**Table 1S** Clinical and Demographic Features of AIE Patients with Antibodies Other than NMDA.

| **Patient number** | **Age (years)** | **Sex** | **Type of antibody** | **OCB** | **Associated tumor** | **Clinical presentation** |
| --- | --- | --- | --- | --- | --- | --- |
| 1 | 56 | Male | CASPR2 | + | None | Behavioral changes, language  problems |
| 2 | 17 | Female | MGLUR5 | + | None | Language problems |
| 1 | 63 | Male | GABAB | - | None | Behavioral changes, memory  deficit, language problems |
| 2 | 69 | Male | LGI1 | - | None | Behavioral changes, memory  deficit |
| 3 | 48 | Female | CASPR2 | - | None | Behavioral changes, language  problems |
| 4 | 79 | Female | MGLUR5 | - | DLBCL | Behavioral changes, memory  deficit |
| 5 | 17 | Female | LGI1 | - | None | Behavioral changes |
